# Differential olfactory dysfunction and nasal tissue pathology in Syrian hamsters infected with SARS-CoV-2 variants

**DOI:** 10.1128/spectrum.00755-25

**Published:** 2025-09-03

**Authors:** Takeshi Saito, Marina Hosotani Saito, Rachel A. Reyna, Satoshi Taniguchi, Kirsten E. Littlefield, Rebecca Cook, Preston Underbrink, Amy Wang, Tomoko Makishima, Slobodan Paessler, Junki Maruyama

**Affiliations:** 1 Department of Pathology, University of Texas Medical Branch, Galveston, Texas, USA; 2 Department of Otolaryngology, University of Texas Medical Branch, Galveston, Texas, USA; 3 Department of Microbiology & Immunology, University of Texas Medical Branch, Galveston, Texas, USA; National Microbiology Laboratory, Winnipeg, Manitoba, Canada

**Keywords:** SARS-CoV-2, animal model, olfactory dysfunction, hamster, offactory histopathology, behavioral test

## Abstract

Severe acute respiratory syndrome coronavirus-2 (SARS-CoV-2), the causative agent of the global coronavirus disease 2019 (COVID-19) pandemic, frequently induces olfactory dysfunction (OD), a symptom that significantly impacts patients' quality of life. Understanding the variability in OD and nasal tissue pathology across different SARS-CoV-2 variants may provide insights as to the mechanisms underlying this symptom and inform therapeutic strategies for COVID-19-related sequelae. This study examines the OD and associated nasal pathology in Syrian hamsters infected with SARS-CoV-2 variants, including Wuhan (WA-1), Alpha, Beta, Gamma, Delta, and Omicron, at 5 days post-infection. Olfactory function was assessed using buried food detection tests. Hamsters infected with the WA-1, Alpha, Beta, and Gamma variants exhibited prolonged discovery times as compared to controls, indicating severe OD. In contrast, those infected with the Delta and Omicron variants showed minimal or mild delays. Pathological examination of the lateral turbinate (LT) revealed severe olfactory epithelial damage in WA-1, Alpha, Beta, Gamma, and Delta variant infections, while the Omicron variant caused minimal damage. Viral antigens were detected in retained and/or sloughed epithelial cells in the LT across all variant infections. These results demonstrate significant differences in OD severity and nasal tissue damage among SARS-CoV-2 variants, providing critical insights for understanding the pathogenesis of this symptom and guiding treatment development for COVID-19 sequelae.

**IMPORTANCE:**

Olfactory dysfunction (OD) is a prominent and distinctive symptom of severe acute respiratory syndrome coronavirus-2 (SARS-CoV-2) infection that significantly affects patients' quality of life. This study reveals that Syrian hamsters infected with different SARS-CoV-2 variants (WA-1, Alpha, Beta, Gamma, Delta, and Omicron) exhibit varying degrees of OD and nasal tissue pathology. Hamsters infected with the WA-1, Alpha, Beta, and Gamma variants experienced significant OD, while those infected with Delta and Omicron showed milder symptoms. Severe epithelial damage in the nasal turbinate was observed in hamsters infected with WA-1, Alpha, Beta, Gamma, and Delta variants, whereas Omicron variant infection resulted in minimal tissue injury. Despite the variability in OD and tissue damage, viral antigen was detected in all infected hamsters. These findings underscore the diverse impact of SARS-CoV-2 variants on olfactory function and nasal tissue pathology, providing important insights into the pathogenesis of OD and guiding potential therapeutic strategies for coronavirus disease 2019-related olfactory sequelae.

## INTRODUCTION

The ongoing global pandemic of coronavirus disease 2019 (COVID-19) is caused by severe acute respiratory syndrome coronavirus-2 (SARS-CoV-2). Although most SARS-CoV-2 infections result in no symptoms or mild flu-like symptoms, some patients experience severe pneumonia, requiring immediate medical care (1). A unique symptom in COVID-19 is olfactory dysfunction (OD), or anosmia (2). COVID-19 is characterized by a high prevalence of anosmia, between 33.9% and 85.6%, which is higher than other viral infectious diseases, occurring with sudden onset and typically short duration (2–6). Anosmia and taste dysfunction may be the sole symptoms without accompanying nasal congestion or rhinorrhea. These chemosensory deficits usually last from several days to around 2 weeks, with most cases improving within 7–10 days (7, 8). The long-term effect of SARS-CoV-2 infection on olfactory function remains unknown. The unique timing and duration of the anosmia provide crucial insights into the potential mechanisms underlying SARS-CoV-2-induced anosmia.

During the first year of the global COVID-19 pandemic, the prototypic strain of SARS-CoV-2 (WA-1) deviated into several variants of concern (VoCs), such as Alpha (B.1.17), Beta (B.1.351), and Gamma (P.1). These VoCs were subsequently almost entirely supplanted by the Delta variant (B1.617.2). By 2021–2022, the Delta variant was replaced by the Omicron variant (B.1.1.529) along with its sub-lineages (e.g., BA.1, BA.2, and BA.5), as well as various recombinant forms like XBBs (9). In 2023, the BA.2.86 sub-variant of Omicron was detected. Subsequently, JN.1, which diverged from the BA.2.86 as the parent strain, was detected, and JN.1 and its sub-lineages (e.g., JN.1.7, KP.2, and KP.3) are the major variants prevalent in the United States in 2024 (10).

The symptoms caused by SARS-CoV-2 variant infections and their severity can vary based on the genetic mutations within each variant, previous infection history, vaccination status, and overall health condition and immune status in patients (11, 12). There have been reports of varying incidence rates of anosmia among these variants (2, 13). However, the severity of anosmia linked to different SARS-CoV-2 variants has not yet been extensively studied in animal models, and the underlying mechanisms remain to be fully elucidated.

Syrian hamsters serve as a suitable animal model for studying SARS-CoV-2 due to their susceptibility to infection with low mortality, significant pathological features reflective of COVID-19 patients, and immunocompetent nature (14–16). Our prior studies have shown that SARS-CoV-2 WA-1-infected hamsters experience significant damage to their olfactory epithelium in the nasal turbinates within three days after infection, a finding supported by behavioral tests indicating olfactory dysfunction (16, 17), demonstrating that hamsters can also be utilized to study for OD caused by SARS-CoV-2 infection.

In this study, using the hamster model, we investigated the degree of anosmia and tissue damage in the olfactory epithelium caused by infection with the prototype SARS-CoV-2 strain (WA-1) and variants of the Alpha, Beta, Gamma, Delta, and Omicron. We employed the buried food detection test to evaluate OD and conducted histopathological analysis to assess tissue damage in the olfactory epithelium.

## MATERIALS AND METHODS

### Cells and viruses

African green monkey kidney Vero E6 cells were grown in Dulbecco’s modified Eagle’s medium (DMEM; Sigma-Aldrich) supplemented with 10% fetal bovine serum (FBS; Gibco), 100 U/mL penicillin, and 0.1 mg/mL streptomycin (Gibco) at 37°C in a 5% CO_2_ incubator. SARS-CoV-2 (USA/WA-1/2020) and Alpha (B.1.1.7; USA/CA-CDC5574/2020), Beta (B.1.351; MD-HP01542/2021), Gamma (P.1; MD-MDH-0841/2021), Delta (B.1.617.2; GNL-751), and Omicron (B.1.1.529; hCoV/EHC_C19_2811C) variants were received from the World Reference Center for Emerging Viruses and Arboviruses at the University of Texas Medical Branch (UTMB) and propagated in Vero E6 cells with DMEM supplemented with 2% FBS. Cell culture supernatants were stored at −80°C until use. All work with infectious SARS-CoV-2 was approved by the Institutional Biosafety Committee at UTMB and performed in BSL-3 facilities at UTMB in accordance with institutional guidelines.

### Virus titration

Lung samples collected from SARS-CoV-2-infected hamsters were homogenized in DMEM supplemented with 2% FBS. Cell culture supernatants of SARS-CoV-2 variants or the lung homogenates were 10-fold serially diluted in DMEM with 2% FBS and inoculated into Vero E6 cells on 96-well plates. Four days after inoculation, the plates were fixed with 10% buffered formalin and stained with 0.25% crystal violet to visualize the cytopathic effect. The 50% tissue culture infectious dose (TCID_50_) value was determined by the Reed and Muench method (18).

### Animal experiments

Five- to six-week-old female Syrian golden hamsters were purchased from Charles River Laboratories according to the previous studies (16, 17). We did not consider sex differences in this study. Seven groups of five hamsters were intranasally inoculated with either 10^5^ TCID_50_ of SARS-CoV-2 WA-1, Alpha, Beta, Gamma, Delta, or Omicron variants diluted in 100 µL of sterile PBS or sterile PBS as mock infection. The body weight of animals was measured daily after the virus infection throughout the study for 5 days. All animals were subjected to a buried food detection test, as previously described (16), to investigate olfactory function at 5 days post-infection (dpi). After the behavioral test, the animals were euthanized to collect nasal turbinates and lungs for histopathological analysis or virus titration. All hamsters were housed in the animal biosafety level-2 (ABSL-2) and ABSL-3 facilities in the Galveston National Laboratory at UTMB. All animal studies are reviewed and approved by the Institutional Animal Care and Use Committee at UTMB and performed with the National Institutes of Health guidelines. This study is reported in accordance with ARRIVE guidelines (https://arriveguidelines.org/arrive-guidelines).

### Behavioral testing

The buried food detection test was performed to examine olfactory function, as previously reported (16). A housing cage with at least 3 cm of bedding was prepared, and a honey-flavored teddy graham (Nabisco) was buried in one corner approximately 1 cm below the surface. A hamster was placed in the corner diagonal from the hidden cookie, the lid was closed, and the time until the hamster found and grasped the cookie was recorded. All hamsters were given a maximum of 300 s to find the buried cookie.

### Histological analysis

Collected nasal turbinates were fixed with 10% buffered formalin for 7 days with a formalin change after 24 h before removal from the BSL-3 facilities. The olfactory bulbs and nasal tissue were extracted from the skull and decalcified with 10% EDTA and embedded in paraffin. The paraffin-embedded tissues were then sectioned at 5 µm and stained with hematoxylin and eosin (HE). The scoring of olfactory epithelium damage was performed, as previously described (16). Briefly, each of the four turbinate zones including nasal septa (S), medial turbinate (MT), dorsal turbinate (DT), and lateral turbinate (LT) was assigned an epithelial damage score: 0—no damage (normal), 1—mild damage (damage reaches only through epithelial layer; basal layer remains untouched), 2—moderate damage (up to 75% of damage reaches basal cells), or 3—severe damage (over 75% of damage reaches basal cells). Scores were averaged between the four reviewers in a blinded manner. The HE-stained sections were imaged using an Olympus IX 71 microscope (Olympus) with a Nikon Digital Sight 1000 digital camera (Nikon) and Nikon NIS-Elements F software (Nikon).

### Immunohistochemistry

The deparaffinized sections were incubated for 20 min in a rice cooker in either 10 mM Tris-1 mM EDTA buffer (pH 9.0) for rabbit anti-Iba1 antibody (FUJIFILM Wako Chemicals, 013-27691) or in Target Retrieval Solution (Dako) for rabbit anti-SARS nucleocapsid protein (N) antibody (NOVUS, NB100-567576). The sections were soaked in 3% hydrogen peroxide solution and then incubated with protein blocking solution (Dako) for 15 min at room temperature. The sections were incubated overnight with anti-Iba1 antibody or anti-SARS N antibody at a dilution of 1:1,000 at 4°C. After washing with PBS, the sections were incubated with SignalStain Boost IHC detection reagent (HRP, Rabbit) (Cell Signaling Technology, 8114S) for 30 min. The immunohistochemical reactions were developed with 3,3′-diaminobenzidine tetrahydrochloride-hydrogen peroxide solution. The sections were lightly stained with hematoxylin and sealed with a coverslip in a mounting medium. The stained sections were examined and images were captured using an Olympus IX 71 microscope with a Nikon Digital Sight 1000 digital camera and Nikon NIS-Elements F software. The scoring of the distribution of viral antigen in each of four turbinate zones was assigned a score using IHC sections labeled with anti-SARS N antibody: 0—0% of immunoreactive positive cells, 1—<20% of immunoreactive positive cells, and 2—>20% immunoreactive positive cells in the olfactory epithelium composing cells. The scoring of the distribution of Iba1-positive macrophages in each of four turbinate zones was assigned a score using IHC sections labeled with anti-Iba1 antibody: 0—<10% of positive reaction, 1–10<30% of positive reaction, and 2—>30% positive reaction in the olfactory epithelium. Each score was averaged between the four reviewers in a blinded manner.

### Histoplanimetry of olfactory glands

The alcian blue stained-sections were used for the detection and histoplanimetry of olfactory glands (Bowman’s glands). The percentage of blue-stained area in the whole lamina propria in three randomly selected regions was calculated and averaged using Colour Deconvolution 1.7 plugin provided in Fiji (National Institutes of Health, Bethesda, MD, USA).

### Statistical analysis

All data were analyzed using GraphPad Prism version 10.2.1 software. For comparison of weight change at each time point, we performed a two-way analysis of variance (ANOVA), followed by Dunnett’s post hoc test. For comparison of virus titer in the lung, behavioral test, histopathological score, and histoplanimetry of olfactory gland, we performed one-way repeated-measures ANOVA, followed by Dunnett’s test. Correlations were determined using a Pearson correlation test. *P* < 0.05 was deemed statistically significant for all analyses.

## RESULTS

### Pathogenicity of SARS-CoV-2 variants and anosmia caused by SARS-CoV-2 variant infection in hamsters

**Fig 1:**
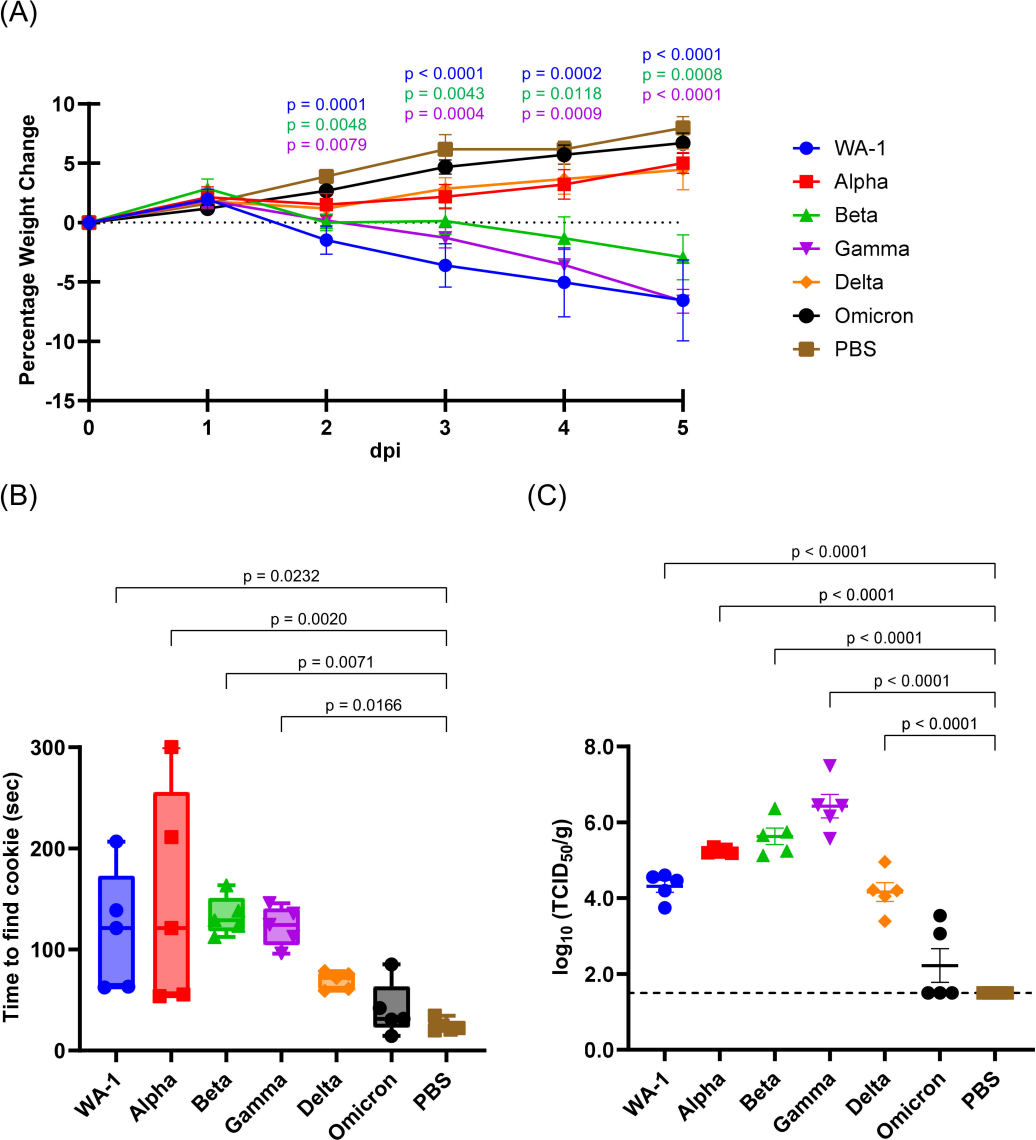
Pathogenicity of SARS-CoV-2 variants and anosmia caused by SARS-CoV-2 variants infection in hamsters. (**A**) Body weight changes of hamsters after SARS-CoV-2 WA-1, Alpha, Beta, Gamma, Delta, or Omicron variant infection. The symbols represent mean group weight changes and the bars represent standard errors. Significant differences between SARS-CoV-2 variants and PBS-inoculated groups at each time point were determined by a two-way ANOVA, followed by Dunnett’s post hoc test. Statistically significant *P* values are indicated in the figure. (**B**) The time required for hamsters to find the hidden cookie was shown in box plots. (**C**) Averages and standard errors of virus titers in the lungs of SARS-CoV-2-inoculated hamsters were plotted. The broken line indicates the limit of detection (<1.50 log_10_ TCID_50_/g). Significant differences in the time required to find the hidden cookie and virus titers compared to PBS-inoculated group were determined by one-way ANOVA, followed by Dunnett’s post hoc test. Statistically significant *P* values are indicated in the figure.

Syrian hamsters were inoculated intranasally with 10^5^ TCID_50_ of SARS-CoV-2 (WA-1) or its variants; Alpha, Beta, Gamma, Delta, and Omicron variants, and monitored for signs of illness and body weight change (Fig. 1A). The WA-1, Beta, and Gamma variants-infected hamsters showed significant weight loss from 2 to 5 dpi compared to the negative control of infection (PBS). Animals infected with Alpha or Delta variants developed minor weight loss at 2 dpi and recovered by 3 dpi. The Omicron variant-infected hamsters did not show body weight loss. None of the SARS-CoV-2-infected animals or mock-infected animals showed any visible signs of illness. At 5 dpi, anosmia was investigated through a buried food detection test (Fig. 1B) (16). The PBS-inoculated animals found the hidden cookies in an average of 24.46 s. Cookie-finding time was significantly extended in WA-1, Alpha, Beta, or Gamma variant-infected hamsters, with average times of 118.55, 148.38, 133.41, and 122.86 s, respectively. One of the Alpha variant-infected animals was unable to find the cookie until the 300 s time limit. In the Omicron-infected animals, there was no significant increase in cookie-finding time compared to PBS inoculation, although one animal required longer than the others, at 85.26 s. After behavioral testing, lung and nasal samples were collected for virus titration and histological analysis. The lung samples of WA-1, Alpha, Beta, Gamma, and Delta variant-infected hamsters had detectable viral titers with mean viral loads measuring 3.57, 4.57, 4.90, 5.65, 3.47 log_10_ TCID_50_/g, respectively (Fig. 1C). Three of the five lung samples from Omicron variant-infected hamsters did not show viral titers above the detection limit (1.50 log_10_ TCID_50_/g), while the average of the two detectable samples was 3.30 log_10_ TCID_50_/g.

### Histopathological analysis of the olfactory epithelium of SARS-CoV-2 variant-infected hamsters

**Fig 2:**
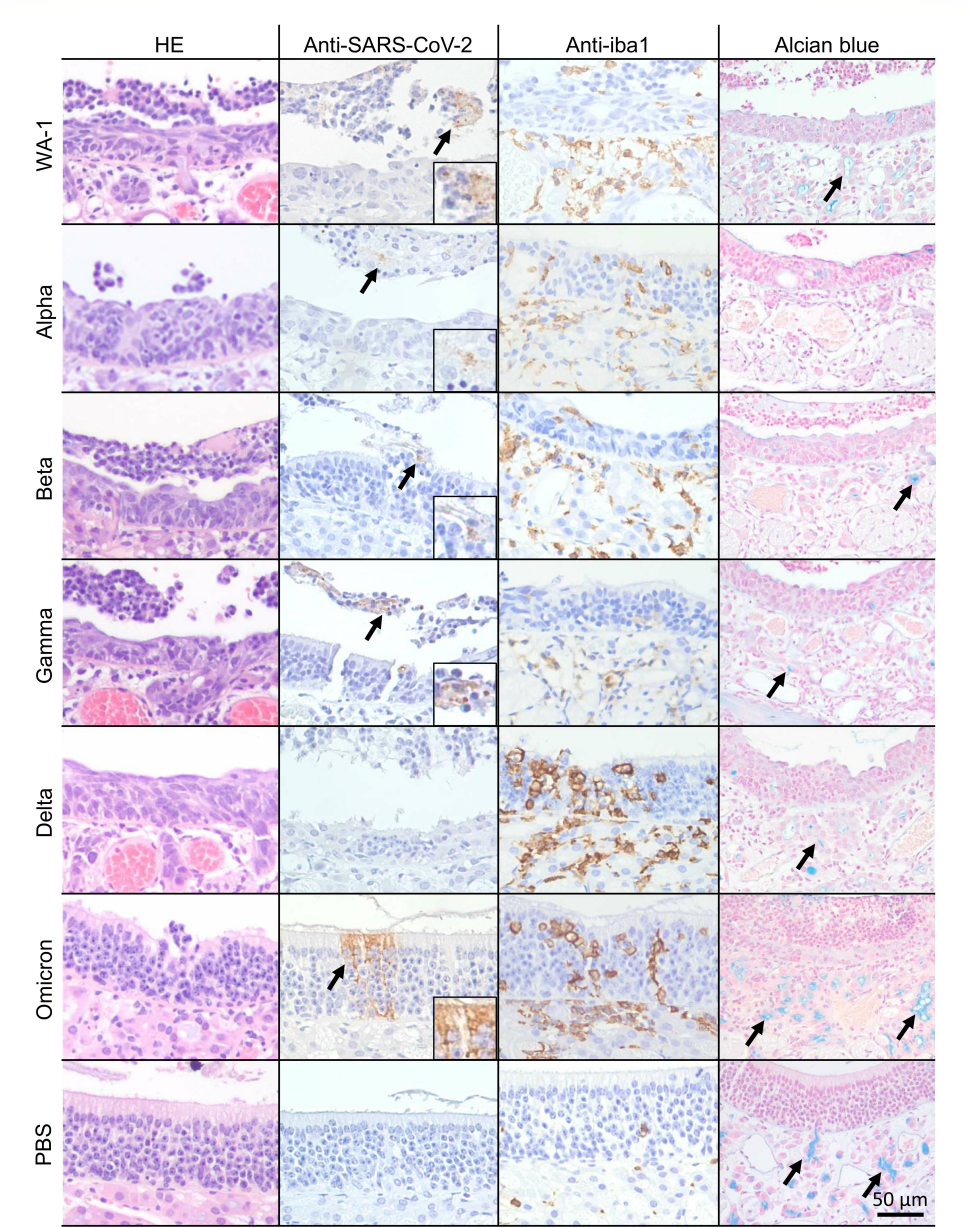
Histological and immunohistochemical changes in the dorsal turbinate of hamsters following SARS-CoV-2 variants infection. (Left) Histological analysis. (Middle left) Immunohistochemistry for SARS-CoV-2 antigen. Arrows indicate viral antigen-positive cells. (Middle right) Immunohistochemistry for Iba1-positive macrophages. Brown-stained cells represent Iba1 antigen-positive cells. (Right) Alcian blue staining. Arrows indicate alcian blue-stained olfactory glands. Scale bar represents 50 µm.

**Fig 3:**
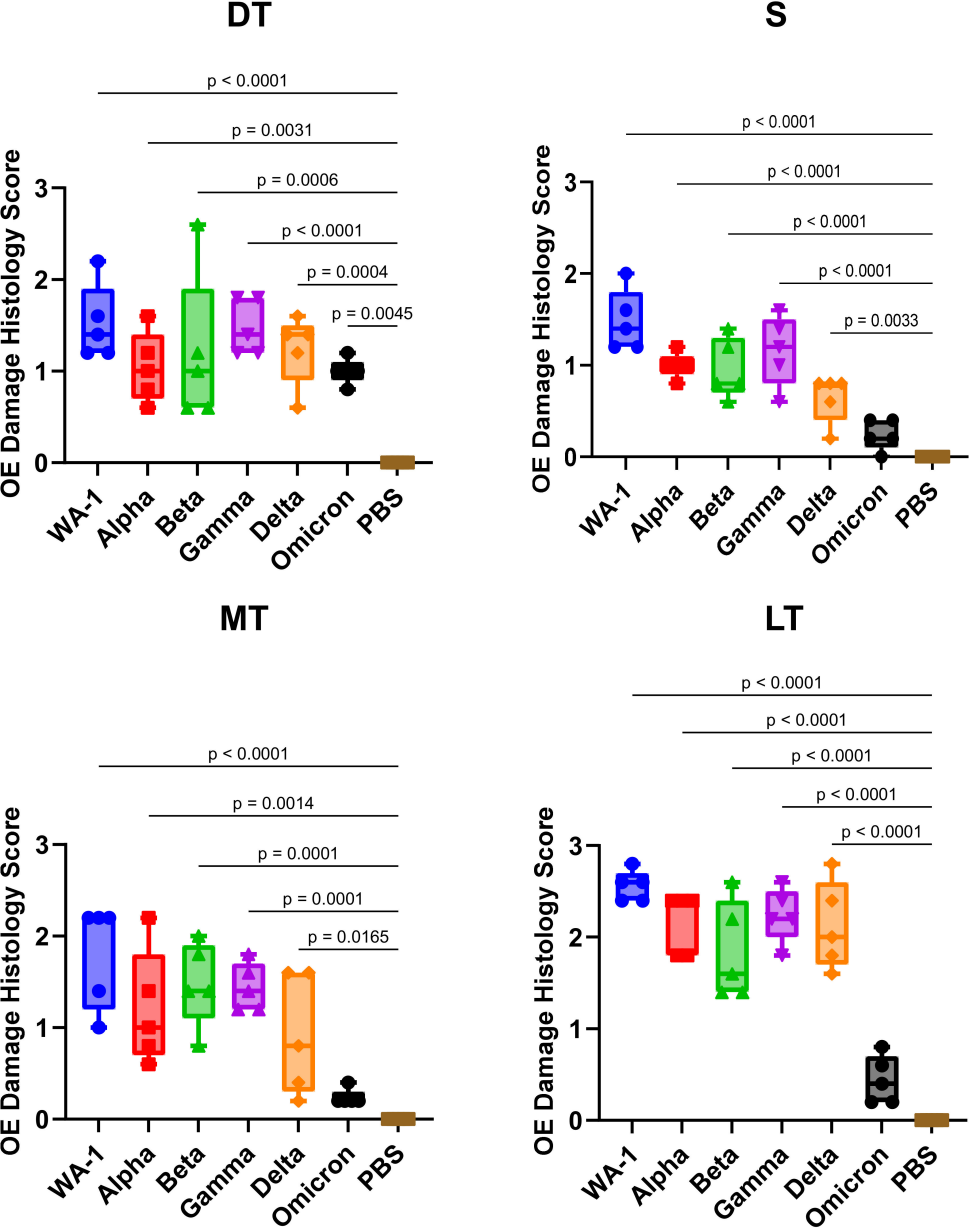
Histopathological score of olfactory epithelium in four nasal turbinate regions: S—nasal septal, MT—medial turbinate, DT—dorsal turbinate, and LT—lateral turbinate. 0—no damage (normal), 1—mild damage (damage reaches only through epithelial layer; basal layer remains untouched), 2—moderate damage (up to 75% of damage reaches basal cells), and 3—severe damage (over 75% of damage reaches basal cells). Scores were averaged among four independent reviewers. Significant differences in histopathological score compared to the PBS-inoculated group were determined by one-way ANOVA, followed by Dunnett’s post hoc test. Statistically significant *P* values are indicated in the figure.

Histopathological analysis was performed on nasal samples collected from SARS-CoV-2 variant-infected hamsters to quantify tissue damage (Fig. 2 and 3; Fig. S1). In WA-1-infected hamsters, nasal tissue damage including degeneration of olfactory epithelial cell alignment, olfactory epithelium cell disruption, and shortening of cilia was severe in S, MT, DT, and LT regions. In particular, the tissue damage in the LT region was the most severe with an average histopathology score of 2.56. In Alpha-infected animals, the tissue damage in S, MT, and DT was less severe, but the damage in LT was also significant with an average histopathology score of 2.16. Tissue damage in the LT region tended to be most severe of the four regions, even in the Beta-, Gamma-, and Delta-infected hamsters with average histopathology scores of 1.84, 2.24, and 2.12, respectively. In Omicron-infected hamsters, there was no or very little tissue damage in the S, MT, and LT regions, though minor tissue damage was observed in the DT region with disruption of olfactory epithelium cell alignment.

### Immunohistochemical analysis of the olfactory epithelium of SARS-CoV-2 variant-infected hamsters

The distribution of viral antigen in the olfactory epithelium of SARS-CoV-2 variant-infected hamsters was investigated using immunohistochemistry (Fig. 2; Fig. S2; Table S1). Viral antigen-positive olfactory epithelial cells were found in both retained and detached olfactory epithelium. In WA-1-infected animals, more virus-infected olfactory epithelial cells were observed in the DT and LT regions compared to the S and MT regions. The Alpha-, Beta-, Gamma-, Delta-, and Omicron variant-infected animals also showed viral antigen-positive cells in the DT and LT regions. In all groups, the number of viral antigen-positive cells in the LT region was similar to or higher than in the DT region. Viral infection of the S region was most commonly observed in the Beta variant-infected group, followed by the Gamma variant-infected group. In the other variant-infected groups, the distribution of the viral antigen-positive cells in this region was absent or minor. In the MT region, more viral antigens were detected in the Beta and Gamma variant-infected groups. In contrast, viral antigens were not detectable in the MT region in Delta and Omicron-infected animals. Interestingly, in the Omicron-infected group, there were areas of viral antigen-positive cells distributed from cilia to epithelial cells in the DT and LT regions. Macrophage infiltration into the lamina propria and the olfactory epithelium was observed in the S, MT, DT, and LT regions to a similar degree in WA-1-, Alpha-, Beta-, and Gamma-infected animals (Fig. 2; Fig. S3; Table S2). On the other hand, macrophage infiltration in the DT region was notable compared to the S, MT, and DT regions in Delta and Omicron-infected animals.

### Histoplanimetric analysis of the olfactory glands in SARS-CoV-2 variant-infected hamsters

**Fig 4:**
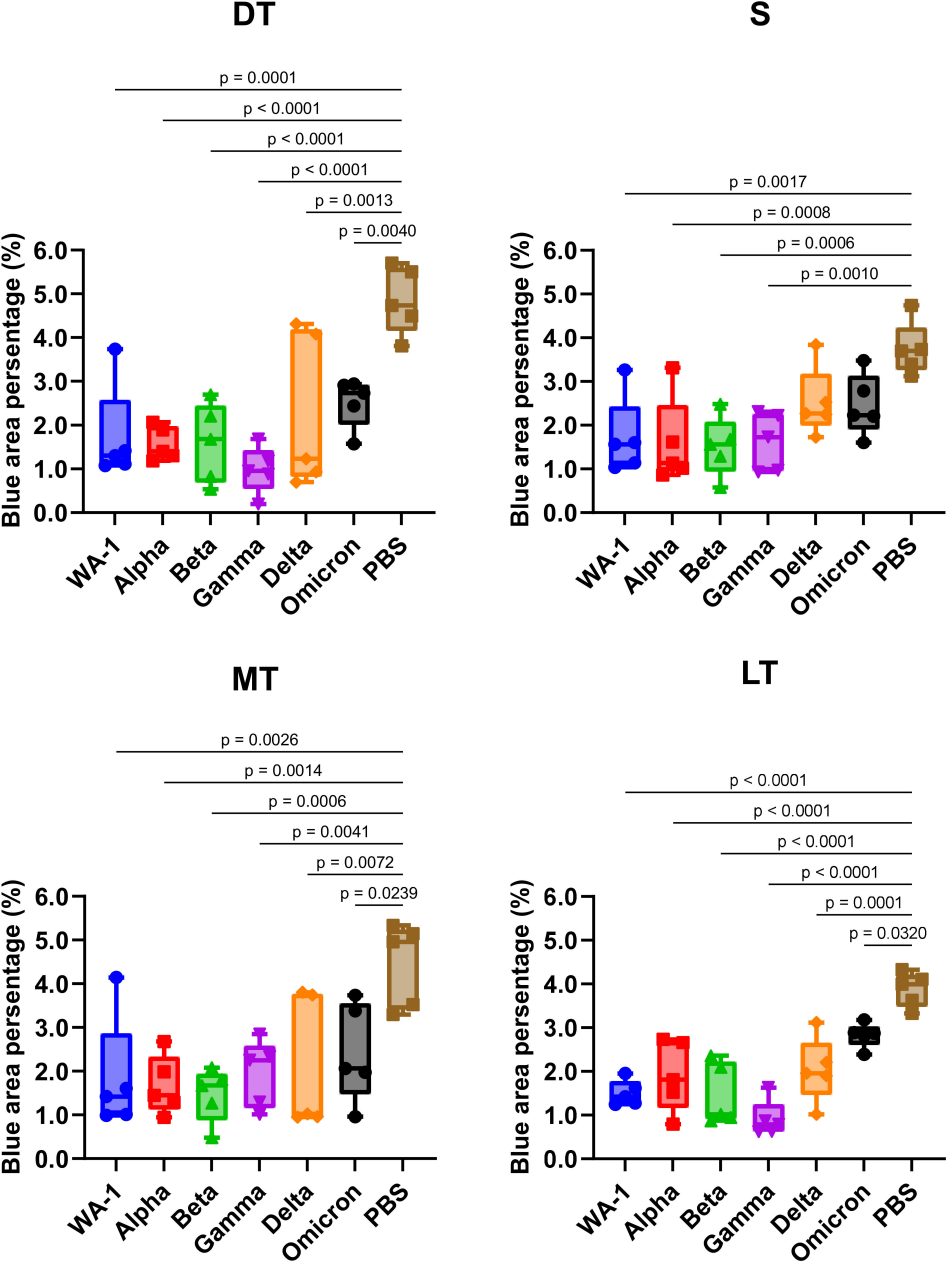
Histoplanimetry of olfactory glands. The alcian blue-stained sections were used for the detection and histoplanimetry of olfactory glands. The percentage of blue-stained area in the whole lamina propria in three randomly selected regions from four nasal turbinate regions: S—nasal septal, MT—medial turbinate, DT—dorsal turbinate, and LT—lateral turbinate was calculated and averaged. Significant differences in histopathological score compared to the PBS-inoculated group were determined by one-way ANOVA, followed by Dunnett’s post hoc test. Statistically significant *P* values are indicated in the figure.

Through secreting mucus, the olfactory glands in the olfactory tissue play a crucial role in maintaining the health of the olfactory epithelium, ensuring proper olfactory function, and protecting against pathogens (19–21). We compared the extent of damage to these glands in hamsters infected with different SARS-CoV-2 variants by measuring the percentage of olfactory gland area stained by alcian blue (Fig. 2 and 4; Fig. S4). In the mock-infected group, the mean blue area percentage was 3.7%, 4.5%, 4.9%, and 3.9% in the S, MT, DT, and LT regions, respectively. In all SARS-CoV-2-infected animals, significant olfactory gland shrinkage ranging from 27% to 80% was observed. In WA-1, Alpha, Beta, and Gamma-infected hamsters, the area of olfactory glands was reduced to less than half, especially in the DT and LT regions of the Gamma-infected group, which lost 80% and 77% of their olfactory glands, respectively.

## DISCUSSION

Among the symptoms associated with COVID-19, anosmia or OD is a unique symptom. There have been reports of varying incidence rates of anosmia among SARS-CoV-2 variant infections. However, the severity of anosmia linked to different SARS-CoV-2 variants has not yet been extensively studied in animal models, and the mechanisms underlying OD caused by SARS-CoV-2 infections remain to be fully elucidated. We previously reported that almost 100% of Syrian hamsters showed OD after SARS-CoV-2 WA-1 infection, with phenotypic confirmation by a buried food detection test (16). No other animal model of OD induced by viral infection has shown such a high rate of OD onset. This hamster model of OD induced by SARS-CoV-2 infection is a powerful tool to reveal the mechanisms underlying post-infection OD caused by viruses through the quantitative assessment of olfactory function.

The buried food detection tests revealed that SARS-CoV-2 WA-1 and Alpha, Beta, Gamma variants caused significant OD in hamsters at 5 dpi, while Delta and Omicron variants caused little to no OD. The hamsters infected with the Alpha variant did not show significant body weight loss, but they did show significant OD, suggesting that there is a minimal relationship between OD and disease severity as indicated by body weight loss. In human cases, the incidence of OD does not necessarily correlate with the incidence of fever or respiratory symptoms, and OD may be the only symptom present (22, 23). Viral replication was observed in the lungs of hamsters infected with the SARS-CoV-2 WA-1, Alpha, Beta, Gamma, and Delta variants. Of note, the viral titer of the Delta variant was as high as that of WA-1, although the Delta variant did not cause significant OD. This is consistent with a previous report indicating that SARS-CoV-2 WA-1 and Gamma variants, but not Delta and Omicron variants, cause significant OD at 3 dpi, which were not associated with viral titer in the lower respiratory tract (24). These results indicated that there is no relationship between anosmia and viral replication within the lower respiratory tract, and that specific mechanisms in the nasal epithelium cause OD in SARS-CoV-2 infection.

The presence of the D614G mutation in the spike protein has been reported to enhance anosmia in COVID-19 patients (25). However, in our olfactory behavioral tests, the SARS-CoV-2 WA-1 lacking this mutation caused significant anosmia in hamsters, while the Delta and Omicron variants with this mutation did not. These results revealed that the D614G mutation is not responsible for the degree of OD. In fact, the global prevalence of Omicron variant-induced OD in adults is estimated to be 3.7%, which is 2–10 times lower than that caused by the Alpha and Delta variants (26). In addition, the sub-lineages of the Omicron variants, BA.2, BQ.1, and BQ.1.1 have been reported to cause significant anosmia in hamsters (27). Thus, the degree of OD is not determined by the D614G mutation, and other differences among SARS-CoV-2 variants are predicted to influence it. The detailed viral factor(s) responsible for OD in SARS-CoV-2 infections can be revealed using reverse genetics in future studies.

The proposed mechanisms of anosmia include damage to olfactory receptor neurons, viral infiltration into the brain, and damage to the supporting cells of the olfactory epithelium related to olfactory function. Tissue damage in the olfactory epithelium is a more likely mechanism for the early onset of OD in COVID-19, since OD is rapidly reversible and viral infection of neurons is not associated with OD (8). Tissue injury of the olfactory epithelium in virus-infected hamsters was indeed associated with OD, and tissue damage in different nasal turbinate regions may be responsible for SARS-CoV-2-induced OD. In WA-1, Alpha, Beta, and Gamma variant infections, tissue damage was severe in all regions, including S and MT. In Delta variant infection, severe damage was seen in DT, MT, and LT, whereas severe tissue damage was seen only in DT in Omicron variant infection. From these findings, we determined that the OD caused by SARS-CoV-2 infection involves tissue damage in the specific nasal turbinate region, particularly in the S and MT regions. A Pearson’s correlation between histopathological score in different nasal turbinate regions and time to find the hidden cookie (Table 1) showed a significant positive correlation. In particular, the strongest correlation was found in MT, suggesting that tissue injury in the MT region leads to OD. In addition, viral antigens were detected in DT and LT regions, but not in S and MT regions, in Delta or Omicron variant infections, suggesting that SARS-CoV-2 variants have different infectivity in the nasal turbinate regions, which may contribute to the degree of tissue damage. In humans and mice, the expression patterns of NADPH: quinone oxidoreductase (NQO1), olfactory marker protein, olfactory cell adhesion molecule, and neural cell adhesion molecule differ depending on the nasal turbinate regions (28–31). Given that NQO1 is highly expressed in the DT region of the nasal turbinate in hamsters (32), the expression of this molecule may play a role in the establishment of infection by the Delta and Omicron strains without causing olfactory abnormalities (33). Macrophage infiltration is also severe in the DT region of hamsters infected with the Delta or Omicron variant, suggesting that inflammation in this region may be less related to OD. This indicates that the spatial distribution of macrophage infiltration, rather than its overall magnitude, may determine its relevance to OD. These findings also raise the possibility that inflammation in distinct olfactory compartments plays varying roles in the development of SARS-CoV-2-induced anosmia.

**TABLE 1:** Pearson’s correlation coefficient (*r*) between histopathology score, histoplanimetry of olfactory glands and time to find the cookie^*a*^

	Histopathology score				Histoplanimetry of olfactory glands			
	S	MT	DT	LT	S	MT	DT	LT
Pearson’s *r*	0.5263	0.6318	0.4049	0.5697	−0.6026	−0.4532	−0.5048	−0.5486
*P* value	0.0012	<0.0001	0.0158	0.0004	0.0001	0.0063	0.002	0.0006

^*a*^ A Pearson correlation was performed for the time taken to find the hidden cookie, the histopathology score of the four individual nasal turbinate regions: S—nasal septal, MT—medial turbinate, DT—dorsal turbinate, and LT—lateral turbinate), and the histoplanmetry of olfactory glands of each turbinate region.

In addition to tissue damage caused by direct virus infection and immune-cell infiltration, olfactory gland shrinkage in each nasal turbinate region caused by SARS-CoV-2 variant infections may also be associated with OD. In hamsters infected with WA-1, Alpha, Beta, and Gamma, which showed significant OD, there was marked olfactory gland shrinkage in all nasal turbinate regions. On the other hand, Delta or Omicron variant-infected animals showed no significant shrinkage in the S region. This observation is consistent with the mild tissue damage in the S region in Delta and Omicron variant infections, suggesting that olfactory gland shrinkage affects the onset of OD in addition to tissue damage in the S region. There were significant negative Pearson’s correlations between olfactory gland area and time to find the hidden cookies (Table 1). The strongest correlation was found in the S region, indicating that olfactory gland atrophy in this region may also be related to OD. Shrinkage of olfactory glands leads to reduced mucus secretion and disruption of the metabolic microenvironment of the olfactory epithelium (33). These findings suggest that the regional pattern of olfactory gland shrinkage, as well as the overall extent of shrinkage, may determine its impact on SARS-CoV-2-induced OD.

Our study reveals that the susceptibility, tissue damage, and immune response in different compartments of the nasal turbinate vary depending on SARS-CoV-2 variants. This variability is characterized by the differential distribution of viral antigens and macrophages, variations in olfactory epithelial damage, and olfactory gland shrinkage, which are related to the degree of OD. This research not only contributes to our knowledge of the impact induced by SARS-CoV-2 infection on the olfactory system but also underscores the importance of variant-specific studies in the ongoing battle against COVID-19.

## SUPPLEMENTAL MATERIAL

Supplemental figures spectrum.00755-25-s0001.pdf Fig. S1 to S4.

Table S1 spectrum.00755-25-s0002.docx Immunohistochemical scoring for SARS-CoV-2 antigen.

Table S2 spectrum.00755-25-s0003.docx Immunohistochemical scoring for Iba1.

ASM does not own the copyrights to Supplemental Material that may be linked to, or accessed through, an article. The authors have granted ASM a non-exclusive, world-wide license to publish the Supplemental Material files. Please contact the corresponding author directly for reuse.
