# Supplementary material for: Differential olfactory dysfunction and nasal tissue pathology in Syrian hamsters infected with SARS-CoV-2 variants
Source: Microbiol Spectr. 2025 Sep 3;13(10):e00755-25. doi: 10.1128/spectrum.00755-25 (PMC12502630; doi:10.1128/spectrum.00755-25)
Supplement: Supplemental figures — Fig. S1 to S4. [file spectrum.00755-25-s0001.pdf]

Fig. S1

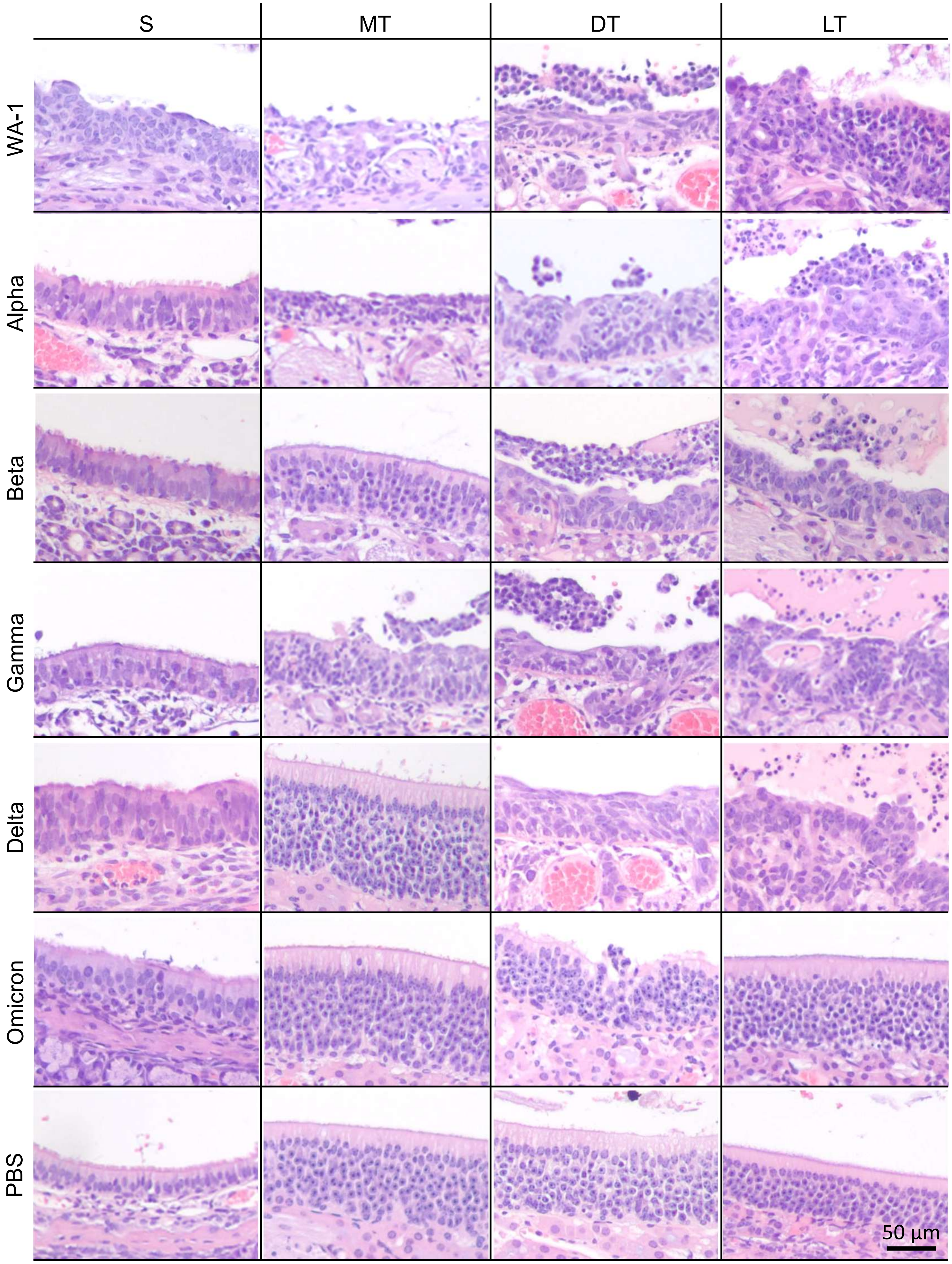

**Fig. S1**

Histological analysis of nasal turbinate in hamsters: S-nasal septal, MT-medial turbinate, DT-dorsal turbinate, LT-lateral turbinate. Scale bar represents 50 μm.

Fig. S2

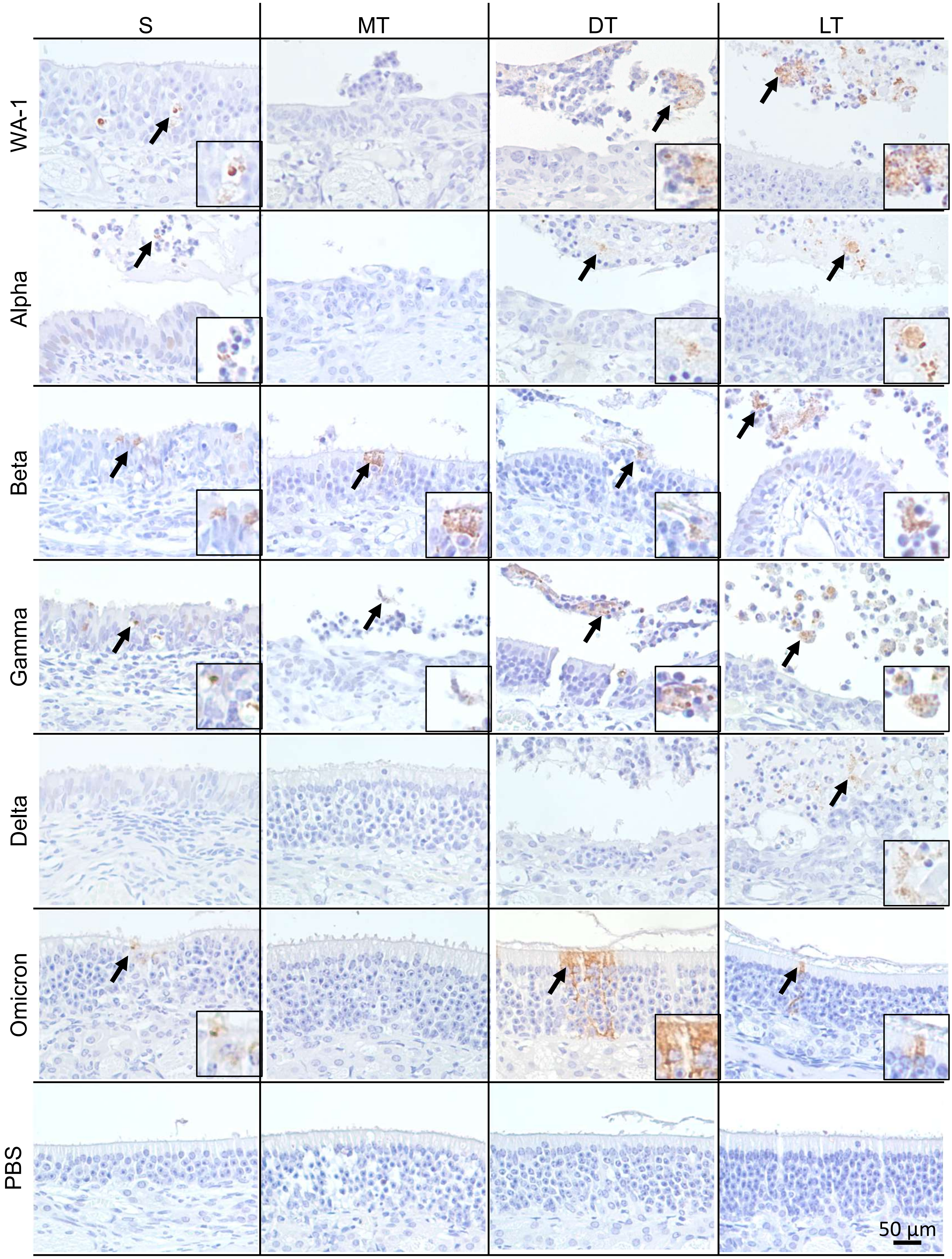

**Fig. S2**

Immunohistochemistry for SARS-CoV-2 antigen of nasal turbinate in hamsters: S—nasal septal, MT—medial turbinate, DT—dorsal turbinate, LT—lateral turbinate. Scale bar represents 50 μm. Arrows indicate viral antigen-positive cells.

Fig. S3

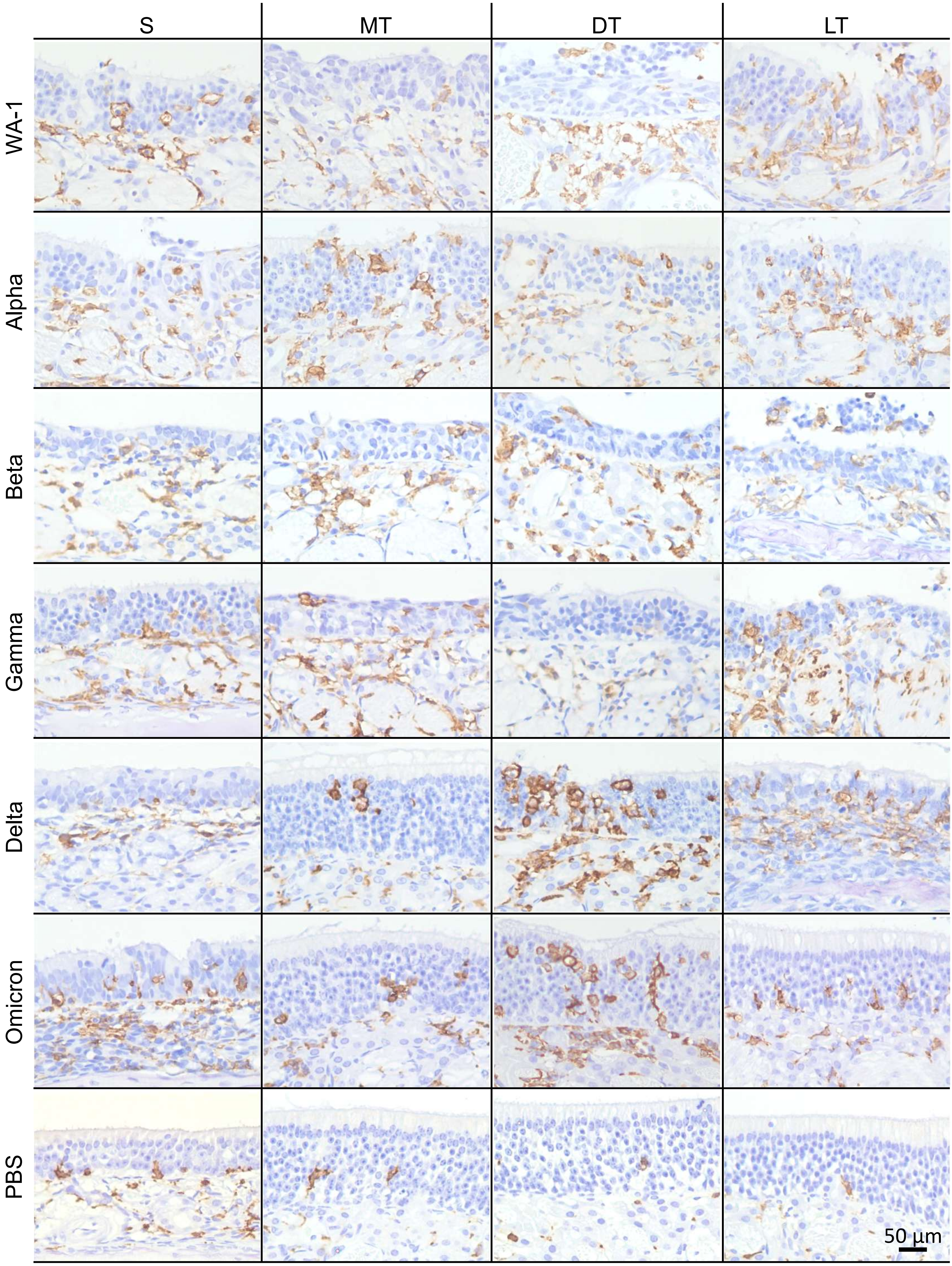

**Fig. S3**

Immunohistochemistry for Iba1-positive macrophages of nasal turbinate in hamsters: S—nasal septal, MT—medial turbinate, DT—dorsal turbinate, LT—lateral turbinate. Scale bar represents 50 μm. Brown-stained cells represent Iba1 antigen-positive cells.

Fig. S4

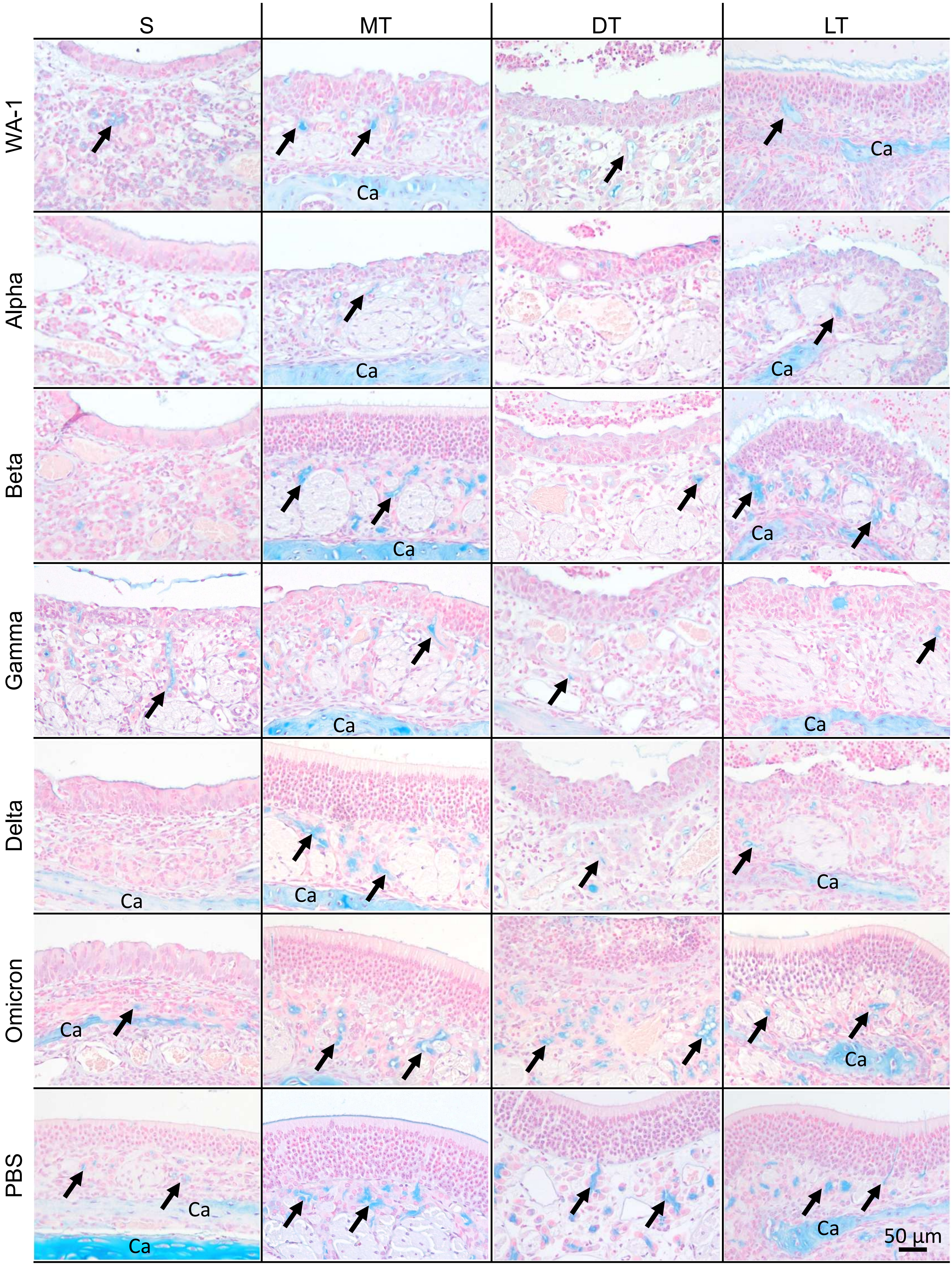

**Fig. S4**

Alcian blue staining of nasal turbinate in hamsters: S—nasal septal, MT—medial turbinate, DT—dorsal turbinate, LT—lateral turbinate, Ca—cartilage. Arrows indicate alcian blue-stained olfactory glands. Scale bar represents 50 μm.
