# Supplementary material for: Differential olfactory dysfunction and nasal tissue pathology in Syrian hamsters infected with SARS-CoV-2 variants
Source: Microbiol Spectr. 2025 Sep 3;13(10):e00755-25. doi: 10.1128/spectrum.00755-25 (PMC12502630; doi:10.1128/spectrum.00755-25)
Supplement: Table S1 — Immunohistochemical scoring for SARS-CoV-2 antigen. [file spectrum.00755-25-s0002.docx]

| Table-S1 Immunohistochemical scoring for SARS-CoV-2 antigen. | | | | | |
| --- | --- | --- | --- | --- | --- |
|  | Animal ID | S | MT | DT | LT |
| WA-1 | 1 | 1 | 1 | 1 | 0 |
|  | 2 | 0 | 0 | 1 | 2 |
|  | 3 | 1 | 1 | 1 | 1 |
|  | 4 | 0 | 0 | 1 | 1 |
|  | 5 | 0 | 1 | 1 | 1 |
|  | Average | 0.4 | 0.6 | 1.0 | 1.0 |
| Alpha | 6 | 0 | 0 | 1 | 1 |
|  | 7 | 0 | 0 | 0 | 1 |
|  | 8 | 0 | 0 | 1 | 1 |
|  | 9 | 1 | 1 | 1 | 1 |
|  | 10 | 0 | 0 | 0 | 1 |
|  | Average | 0.2 | 0.2 | 0.6 | 1.0 |
| Beta | 11 | 1 | 1 | 1 | 1 |
|  | 12 | 1 | 1 | 1 | 1 |
|  | 13 | 0 | 0 | 1 | 1 |
|  | 14 | 1 | 1 | 1 | 2 |
|  | 15 | 1 | 1 | 1 | 2 |
|  | Average | 0.8 | 0.8 | 1.0 | 1.4 |
| Gamma | 16 | 1 | 0 | 2 | 2 |
|  | 17 | 1 | 1 | 1 | 1 |
|  | 18 | 0 | 1 | 1 | 2 |
|  | 19 | 0 | 1 | 1 | 1 |
|  | 20 | 1 | 1 | 0 | 2 |
|  | Average | 0.6 | 0.8 | 1.0 | 1.6 |
| Delta | 21 | 0 | 0 | 1 | 1 |
|  | 22 | 0 | 0 | 1 | 1 |
|  | 23 | 1 | 0 | 0 | 1 |
|  | 24 | 0 | 0 | 0 | 1 |
|  | 25 | 0 | 0 | 1 | 2 |
|  | Average | 0.2 | 0.0 | 0.6 | 1.2 |
| Omicron | 26 | 0 | 0 | 1 | 0 |
|  | 27 | 0 | 0 | 1 | 1 |
|  | 28 | 1 | 0 | 1 | 1 |
|  | 29 | 0 | 0 | 0 | 1 |
|  | 30 | 0 | 0 | 1 | 1 |
|  | Average | 0.2 | 0.0 | 0.8 | 0.8 |
| PBS | 31 | 0 | 0 | 0 | 0 |
|  | 32 | 0 | 0 | 0 | 0 |
|  | 33 | 0 | 0 | 0 | 0 |
|  | 34 | 0 | 0 | 0 | 0 |
|  | 35 | 0 | 0 | 0 | 0 |
|  | Average | 0.0 | 0.0 | 0.0 | 0.0 |
| 0 | 0% positive reaction in the epithelial cells or pealed cells | | | | |
| 1 | 0<-20% positive reaction in the epithelial cells or pealed cells | | | | |
| 2 | more than 20% positive reaction in the epithelial cells or pealed cells | | | | |
